# Ion beam-generated surface ripples: new insight in the underlying mechanism

**DOI:** 10.1186/1556-276X-8-336

**Published:** 2013-07-26

**Authors:** Tanuj Kumar, Ashish Kumar, Dinesh Chander Agarwal, Nirnajan Prasad Lalla, Dinakar Kanjilal

**Affiliations:** 1 Inter-University Accelerator Centre, Aruna Asaf Ali Marg, New Delhi, 110067, India; 2 Indian Institute of Technology Delhi Hauz-Khas, New Delhi, 110016, India; 3 UGC-DAE Consortium for Scientific Research, University Campus, Khandwa Road, Indore, 452017, India

**Keywords:** Ion beam, Ripples, TEM, AFM, 81.16.Rf, 68.35.Ct, 68.49.Sf, 79.20.Rf

## Abstract

A new hydrodynamic mechanism is proposed for the ion beam-induced surface patterning on solid surfaces. Unlike the standard mechanisms based on the ion beam impact-generated erosion and mass redistribution at the free surface (proposed by Bradley-Harper and its extended theories), the new mechanism proposes that the incompressible solid flow in amorphous layer leads to the formation of ripple patterns at the amorphous-crystalline (a/c) interface and hence at the free surface. Ion beam-stimulated solid flow inside the amorphous layer probably controls the wavelength, whereas the amount of material transported and re-deposited at a/c interface control the amplitude of ripples.

## Background

Fabrication of self-organized nano-structures over solid surfaces using energetic ion beam irradiation has received a remarkable attention in the last couple of decades. It is an elegant and cost-effective single-step approach over lithographic methods for device fabrication. In general, a uniform ion irradiation of solid surfaces for intermediate energies (10^2^ to 10^4^ eV) causes a self-organized topographic pattern of ripples, holes, or dots [1-4]. On the other hand, irradiation with higher energies (10^6^ to 10^8^eV) causes the phase transformations [5]. In 1988, the first analytical approach to study the surface patterning was given by Bradley and Harper (BH) [6] on the basis of two competing processes: the destabilizing effect of curvature dependent roughening and the stabilizing effect of surface diffusion. Further theoretical refinements of BH's model have been proposed to underline the secondary effect of local curvature-dependent sputtering, ion beam-induced smoothing, and hydro-dynamical contribution [7,8]. BH's linear and its extended models explain many experimental observations but suffered many limitations also [9-11]. Investigations by Madi et al. [11] and Norris et al. [12] showed that the ion impact-induced mass redistribution is the prominent cause of surface patterning and smoothening for high and low angles, respectively. Castro et al. [13,14] proposed the generalized framework of hydrodynamic approach, which considers ion impact-induced stress causing a solid flow inside the amorphous layer. They pointed out that the surface evolution with ion beam is an intrinsic property of the dynamics of the amorphous surface layer [15]. All above experimental findings and their theoretical justification raise questions on lack of a single physical mechanism on the origin and evolution of ripples on solid surface.

In this work, we propose a new approach for explaining all ambiguity related to the origin of ripple formation. We argue that amorphous-crystalline interface (a/c) plays a crucial role in the evolution of ripples. We have shown that the ion beam-induced incompressible solid flow in amorphous layer starts the mass rearrangement at a/c interface which is responsible for ripple formation on the free surface rather than earlier mentioned models of curvature-dependent erosion and mass redistribution at free surface.

## Presentation of the hypothesis

**Figure 1:**
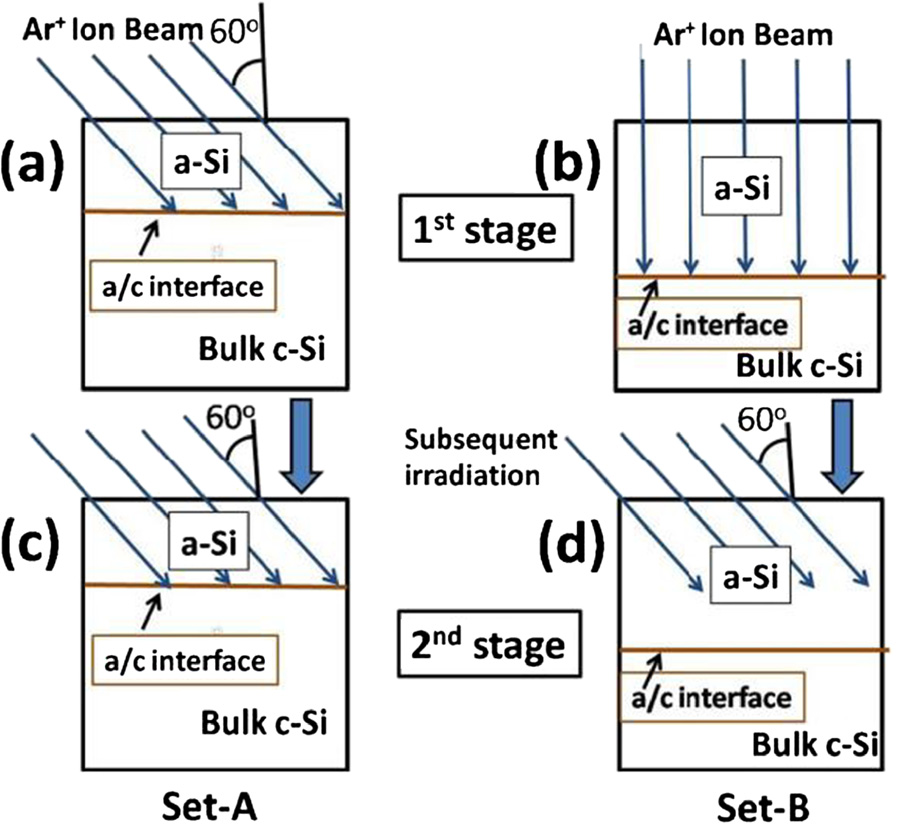
**Schematic view of 50 keV Ar**^**+ **^**ion beam irradiation.** For first stage (to prepare two deferent depth locations of a/c interface) at an angle of **(a)** 60° and **(b)** 0°, with respect to surface normal; second stage irradiation (for fabrication of ripples) at an angle of 60° named as **(c)** set A and **(d)** set B.

In order to study the role of a/c interface in surface patterning of Si (100) surface during irradiation, we performed a series of experiments by preparing two sets of samples with different depth locations of a/c interface. The variation in depth location of a/c interface is achieved by irradiating the Si surface using 50 keV Ar^+^ ion at a fluence of 5 × 10^16^ ions per square centimeter (for full amorphization) at different angles of incidence, viz, 60° (sample set A) and 0° (sample set B) with respect to surface normal. The depth location of a/c interface would be higher in set B samples as compared to set A samples due to higher projected ion range for 0° as compared to 60° ion beam irradiation. Figure 1a,b shows the schematic view for ion beam-stimulated damage range for off-normal incidence of ion beam at 60° (named as set A) and normal incidence (named as set B), respectively. Subsequently, to grow ripples in the second stage of irradiation, both sets of samples were irradiated at an angle of 60° wrt surface normal using 50 keV Ar^+^ ion beam, as shown in Figure 1c,d. For the set A samples, ion beam-stimulated damage effect will reach at a/c interface in the second stage irradiation while it remains inside the amorphous layer for set B samples due to deeper depth location of a/c interface. To have detailed experimental data, a number of samples were prepared by varying fluence from 3 × 10^17^ to 9 × 10^17^ ions per square centimeter for each set. During the irradiation, the base pressure of chamber was maintained at approximately 10^−7^ mbar. The ion beam current density was kept constant at 15 μA/cm^2^. The beam was scanned uniformly over an area of 10 mm × 10 mm by electromagnetic beam scanner. After irradiation, the samples were analyzed by Nano Scope IIIa atomic force microscope (AFM; Bruker AXS Inc, Madison, WI, USA) under ambient conditions in tapping mode. Cross-sectional transmission electron microscopy (XTEM) was carried using a Tecnai-G2-20 TEM (FEI, Hillsboro, OR, USA) facility operating at 200 kV. The cross-sectional specimens for TEM study were prepared by Ar ion beam milling at 4 kV/20 μA and at an angle of 4° with respect to the sample surface.

## Testing the hypothesis

**Figure 2:**
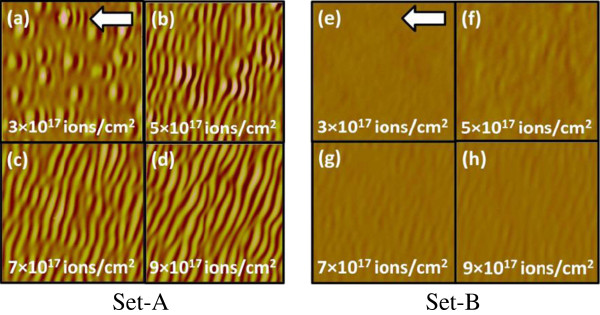
**AFM images for the 50 keV Ar**^**+**^-**irradiated set A and set B samples at an angle of 60°.** At the fluences of 3 × 10^17^**(a,e)**, 5 × 10^17^**(b,f)**, 7 × 10^17^**(c,g)**, and 9 × 10^17^ ions per square centimeter **(d,h)**, respectively. The arrows in the figures indicate the projection of ion beam direction on the surface.

**Figure 3:**
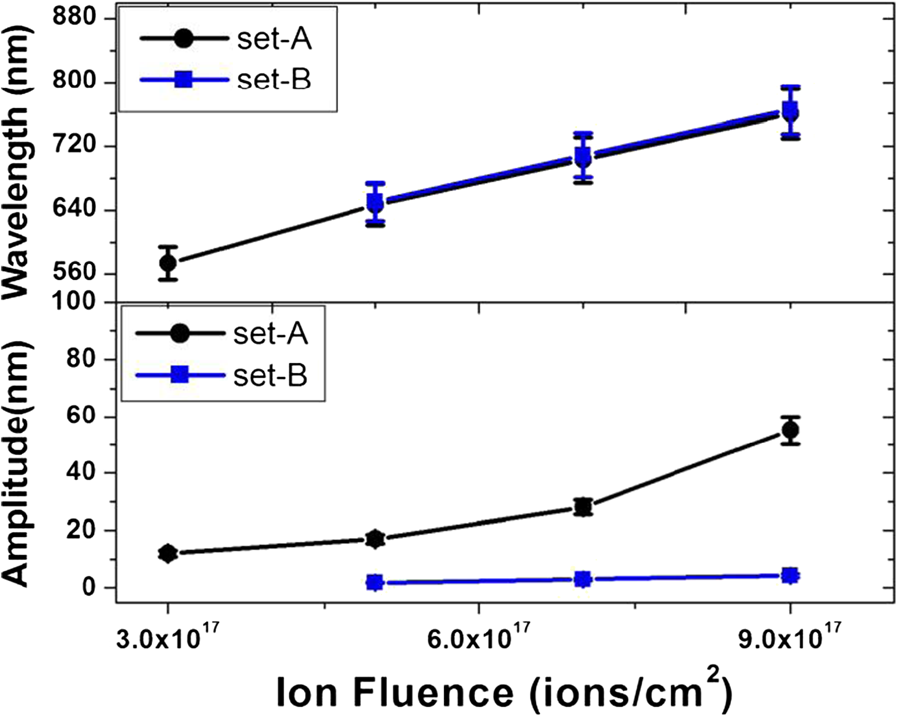
Variation of wavelength and amplitude of ripples for set A and set B samples with ion beam fluence.

AFM characterization was carried out on all samples after each irradiation step. After first irradiation, the average RMS roughness for both sets of the samples was nearly similar (0.5 ± 0.1 and 0.6 ± 0.1 nm). In the second stage, all samples were irradiated by a stable 50 keV Ar^+^ at same angle of incidence (60°) for all fluences. Figure 2a,b,c,d, and e,f,g,h shows the AFM images for set A and set B samples after the second stage irradiation at the fluences of 3 × 10^17^, 5 × 10^17^, 7 × 10^17^, and 9 × 10^17^ ions per square centimeter, respectively. It was found that for set A, the wavelength and amplitude were increasing with increase in irradiation fluence (as shown in Figure 3). For set B samples, the average wavelengths of ripples were nearly same as that of set A samples at corresponding fluences. However, the observed average amplitudes of ripples are about one order less in magnitude for set B as compared to those for set A since the only difference between two sets of samples was the depth location of a/c interfaces. If the evolution ripples were based on curvature-dependent sputtering and surface diffusion, we should have got ripples of identical dimensions for corresponding equal fluence in both sets of samples. Despite similar initial surface morphology of both sets of samples after first stage of irradiation, the observation of similar wavelength and lower amplitude of ripples in set B samples as compared to set A samples casts doubt on the validity of Bradley-Harper and its extended theories. It can be emphasized here that we repeated complete set of experiment with two different ion beams and at different energies (Ar at 50 keV and Kr at 60 keV). And in all cases, the observed trend was similar. To the best of the authors' knowledge, there is no existing model which could physically explain this anomaly. The prominent role of the a/c interface in formation of ripples is established in this work.

**Figure 4:**
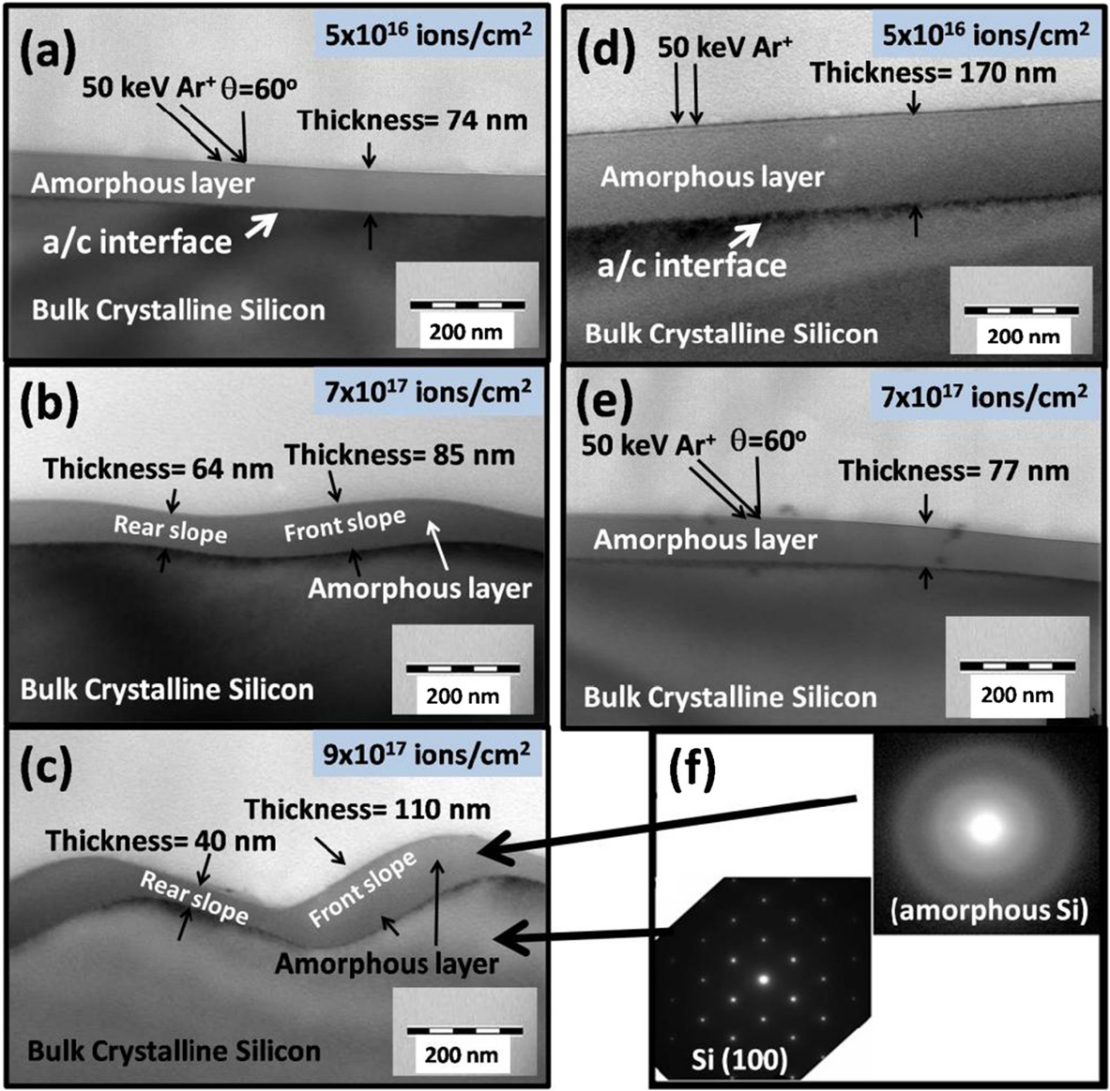
**X-TEM images of 50 keV Ar**^**+**^-**irradiated set A samples.** At the fluences of **(a)** 5 × 10^16^, **(b)** 7 × 10^17^, **(c)** 9 × 10^17^ ions per square centimeter, and set B samples **(d)** 5 × 10^16^ (for normal incidence) and **(e)** 7 × 10^17^ ions per square centimeter. SAED pattern for the amorphized and bulk crystalline regimes is in **(f)**.

Figure 4a,b,c shows XTEM images for set A samples corresponding to irradiation fluences of 5 × 10^16^ (after first irradiation), 7 × 10^17^, and 9 × 10^17^ ions per square centimeter, respectively. Similarly, Figure 4d,e images are for set B samples irradiated at fluences of 5 × 10^16^(after first irradiation) and 7 × 10^17^ ions per square centimeter, respectively. For the set A samples (Figure 4a), it was observed that top amorphous layer has a uniform thickness of about 74 nm which after irradiation at 7 × 10^17^ ions per square centimeter, results in ripple formation. From the XTEM images and using grid line method [16], it was found that during the rippling processes, the overall cross-sectional area of amorphous layer remains constant which validates the condition of incompressible solid mass flow inside the a-Si layer [13,14]. For the set B samples, the initial a-Si layer thickness was found to be 170 nm, as shown in Figure 4d. Interestingly, the thickness of a-Si was found to be decreased to 77 nm for the subsequent irradiated sample for the fluence of 7 × 10^17^ ions per square centimeter, (Figure 4e). Observed ripple dimensions for all samples measured from XTEM were consistent with AFM data. Selected area diffraction (SAED) pattern taken on both sides of a/cinterface confirmed the amorphized and bulk crystalline regions, as shown in Figure 4f.

## Implication of the hypothesis

To physically understand the underlying mechanism, we considered a radical assumption that the formation of ripples is initiated at a/c interface due to the erosion and re-deposition of Si atoms under the effect of solid flow. Due to incompressible nature of this solid mass flow inside amorphous layer, structures formed at the a/c interface reciprocate at the top surface. Similar process of ripple formation on sand (ripples caused by air flow on sand dunes, etc.) has been well observed and studied [17,18]. Here, we assume that the rearrangement of Si atoms is taking place at the a/c interface due to solid flow inside damaged layer, which controls the process of ripple formation. In the case of set A samples, the rearrangement of Si atoms at the a/c interface starts instantaneously with second stage irradiation as the ion range is equal to depth of a/c interface. However, for set B samples, second stage irradiation results in surface erosion before the ion beam effect reach at a/c interface. Thus, the process of mass rearrangement at a/c interface lags behind in set B samples as compared to set A samples. This fact was confirmed by the formation of ripples with appreciable average amplitude (23 nm) and wavelength (780 nm) observed at still higher fluence of 1.5 × 10^18^ ions per square centimeter. Therefore, amplitude is less in magnitude in set B samples as compared to set A samples at corresponding fluences. Since the ion beam parameters are identical in the second stage of irradiation, so the solid flow would be identical in both set of samples. This solid flow is probably responsible for the similar wavelength of ripples for both set of samples. Castro et al. [13,14] and Kumar et al. [16] have also discussed role of solid flow for surface rippling. As already discussed, our AFM and XTEM results could not be explained by existing models of BH and its extended theories, where they consider it only surface effect. The role of a/c interface has not been considered in the formation of ripples on solid surfaces by earlier groups [6,12,13]. By considering ripple formation as an a/c interface-dependent process, all phenomena like ripple coarsening, propagation, etc., can be correlated.

## Conclusions

In conclusion, by designed experiments and theoretical modeling, a new approach for explaining the origin of ripple formation on solid surface has been proposed. Formation of ripples at top surface is a consequence of mass rearrangement at the a/c interface induced by incompressible solid flow inside the amorphous layer. The control parameter for ripple wavelength is solid flow velocity, while that for the amplitude is amount of silicon to be transported at the interface.

## Competing interests

The authors declare that they have no competing interests.

## Authors’ contributions

TK designed and performed the experiments, and analyzed the results. AK helped in the analysis of results as well as in writing the manuscript. DC helped during the irradiation of samples and in XTEM analysis. NP performed the XTEM measurement. DK participated and contributed in the design of study and coordination. All authors read and approved the final manuscript.
